# Crystal structure of *E. coli* arginyl-tRNA synthetase and ligand binding studies revealed key residues in arginine recognition

**DOI:** 10.1007/s13238-013-0012-1

**Published:** 2014-01-30

**Authors:** Kelei Bi, Yueting Zheng, Feng Gao, Jianshu Dong, Jiangyun Wang, Yi Wang, Weimin Gong

**Affiliations:** Laboratory of Non-coding RNA, Institute of Biophysics, Chinese Academy of Sciences, Beijing, 100101 China

**Keywords:** arginyl-tRNA synthetase, amino acyl-tRNA synthetase, isothermal titration calorimetry, site-directed mutation, X-ray crystal structure, *E. coli*

## Abstract

The arginyl-tRNA synthetase (ArgRS) catalyzes the esterification reaction between L-arginine and its cognate tRNA^Arg^. Previously reported structures of ArgRS shed considerable light on the tRNA recognition mechanism, while the aspect of amino acid binding in ArgRS remains largely unexplored. Here we report the first crystal structure of *E. coli* ArgRS (eArgRS) complexed with L-arginine, and a series of mutational studies using isothermal titration calorimetry (ITC). Combined with previously reported work on ArgRS, our results elucidated the structural and functional roles of a series of important residues in the active site, which furthered our understanding of this unique enzyme.

**Electronic supplementary material:**

The online version of this article (doi:10.1007/s13238-013-0012-1) contains supplementary material, which is available to authorized users.

## Introduction

Aminoacyl-tRNA synthetase (aaRS) serves to generate the raw materials for protein synthesis—aminoacyl-tRNA in organisms of all complexities. aaRS catalyzes the esterification reaction between the amino acid carboxyl group and the 2′ or 3′ end hydroxyl group on the corresponding tRNA. The highly specific recognition of amino acid and its cognate tRNA is a critical aspect of the enzyme, the malfunction of which may severely impact cellular survival. Understanding the mechanism of amino acid binding is also beneficial to the ongoing efforts of protein expression with non-natural amino acids (Hendrickson et al., 2004; Wang and Schultz, 2005; Sinkeldam et al., 2010; Wang et al., 2012). Namely, natural aaRSs are ideal templates for creating new aaRSs that recognize side chain moieties beyond the pool of 20 natural amino acids.

Being such an essential enzyme for medicine and protein engineering, aaRSs received extensive research in the past two decades (Martinis et al., 1999; Woese et al., 2000). The binding and catalytic mechanisms of most aaRSs are now well understood, based on which the 20 aaRSs are categorized into two classes. As of biochemical functions, the 20 aaRSs differ only in the recognition of cognate amino acid side chain and tRNA anti-codon, whereas the primary and tertiary structures of aaRSs are highly diverse. Among aaRSs from different species, very low sequence similarity exists between organisms even closely related evolutionarily, indicative of convergent evolution.

A member of the class I aaRS, the arginyl-tRNA synthetase (ArgRS) possesses several distinct characteristics. Prior to the formation of the arginyl-tRNA bond, the activation of arginine by ATP requires the binding of tRNA to the enzyme, which is only observed in GlnRS and GluRS (Mehler and Mitra, 1967; Mitra and Smith, 1969; Kern and Lapointe, 1980). ArgRSs of many organisms lack the canonical KMSK motif which constitutes the active site of all other class I aaRSs (Zhou et al., 1997). There are six sets of codons encoding arginine, which poses considerable challenge to ArgRS insofar as identifying cognate tRNA via matching the anti-codons. Consequently, ArgRS utilizes several highly conserved basis on the D-loop region of tRNA as additional identity elements. To better recognize these identity elements, ArgRS possesses an N-terminal domain of roughly 100 amino acids—the Add1 domain, which is unique among aaRSs.

Hitherto, several research groups have reported the crystal structures of ArgRS from three species. The 3D structures of yeast ArgRS-L-arginine complex and the ternary complex of ArgRS-L-arginine-tRNA^Arg^ shed considerable light on the mechanism of tRNA recognition in ArgRS (Cavarelli et al., 1998; Delagoutte et al., 2000). The recognition of anti-codons by the enzyme opens the active site of ArgRS, which in part rationalized the dependence on tRNA binding for arginine activation. The crystal structure of the *Thermus thermophilus* ArgRS (ttArgRS) apoenzyme, reported by Shimada et al., further accounted for the critical role of the identity element A20 on tRNA recognition (Shimada et al., 2001). Konno and co-workers determined the structure of ArgRS-tRNA^Arg^ complex from *Pyrococcus horikoshii* (pyroArgRS). The atomistic model of the ATP-bound complex, constructed based on the pyroArgRS structure, depicted the detailed mechanism of arginine activation in the presence of ATP (Konno et al., 2009).

While most research efforts focused on tRNA recognition and ATP-PPi exchange, the details of arginine binding are largely unexplored. Although Cavarelli et al. identified three highly conserved residues in the binding pocket of yArgRS, the thermodynamic contributions of these and other surrounding residues to the overall binding affinity remain elusive. In addition, ArgRS from *E. coli* (eArgRS), which shares only 29% sequence similarity with yArgRS, has no reported crystal structure. Herein, we report a 2.9 Å structure of the eArgRS-L-arginine complex (PDB ID: 4OBY) and a series of mutational studies of the enzyme active site. Our results showed that not all the conserved residues are critical for substrate binding. The thermodynamic details of the ligand binding reaction provided a mechanistic view of how ArgRS achieved high affinity and specificity towards L-arginine. As an enzyme from the model organism *E. coli*, an atomistic model of eArgRS would help further understand the structure-function relationship of ArgRS and provide structural guidance for future modifications of ArgRS in protein engineering.

## Results

### The overall structure of *E. coli* ArgRS

The refined structure contains the whole sequence except residues Q178–A187, D203–E204 and the C-terminal M577 (Fig. 1A). It can be divided into five domains. The crystallographically invisible residues (178–187 and 203–204) are part of the insertion domain 1 (Ins1) as defined by Cavarelli and co-workers (Cavarelli et al., 1998). *E. coli* ArgRS contains the N-terminal additional domain (Add1, residues 1–112), the catalytic domain (113–382) including two insertion domains (Ins1, residues 164–221, and Ins2, residues 258–311), and the C-terminal additional domain (Add2, 383–577). A well-ordered L-arginine binds in the active site of the catalytic domain. Although 2.5 mmol/L ATP was always present in the crystal growth solution, ATP molecule is not visible in the electronic density map. The difficulty in obtaining a complex structure of ArgRS with ATP stably bound has been noted by several research groups and was attributed to the following two reasons: 1) the presence of both tRNA^Arg^ and L-arginine are prerequisite for ATP binding (Rath et al., 1998); 2) a lack of sufficient hydrophobicity and certain critical residues at the ATP binding site may have prevented the stable binding of ATP and ATP-analog (Konno et al., 2009).

**Figure 1:**
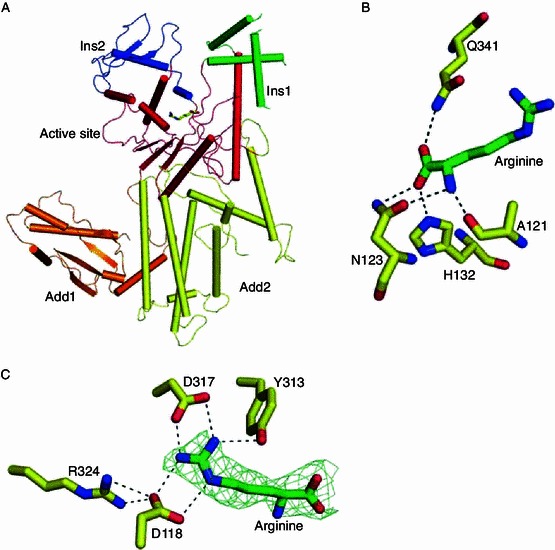
**Overall structure of the eArgRS-L-arginine complex and the detailed structure of the binding site**. (A) Overall structure of the eArgRS-L-arginine complex. The five domains previously identified by Cavarelli et al. are highlighted in different colors. (B) Four residues form hydrogen bonds with the main chain atoms of arginine. (C) Three conserved residues help recognize and hold the side chain guanidinium moiety. The omit map of L-arginine was generated at σ = 2.5

### L-arginine binding and recognition

In the refined structure, a well-ordered arginine molecule is present in the active site (Fig. 1A). The active site of eArgRS consists of a Rossman fold (α11, α12) and one strand (β5, Supplemental Figure). L-arginine binds tightly to the enzyme via a network of hydrogen bonds and salt bridge interactions. The main chain atoms of arginine are recognized by a series of residues strictly conserved in ArgRS, e.g. N123 and Q341. The β-bulge between the important strand β5 and helix α5 encompasses the main chain atoms of L-arginine. Three residues in the region (A121, N123 and H132) formed hydrogen bonds to the main chain atoms of L-arginine and adopted similar orientations as observed in yeast structures (Asn153 and Gln375 of yArgRS), independent of tRNA binding (Cavarelli et al., 1998; Delagoutte et al., 2000). Our structure therefore confirms the discovery from yArgRS structures that tRNA is not required for L-arginine binding (Fig. 1). The side chain recognition motif, which dictates the specificity of the enzyme, contains three conserved residues (D118, Y313, D317) making direct H-bonds (or salt bridges) to the guanidinium moiety of arginine. Another residue R324 formed two hydrogen bonds with D118, stabilizing the orientation of the latter.

We mutated the four aforementioned residues to alanine and generated four stable mutant ArgRSs (D118A, Y313A, D317A and R324A). The binding thermodynamics between ArgRS and L-arginine, determined via ITC, confirmed that these four residues were important for the recognition of arginine (Fig. 1B and 1C). Consistent with our structural data, the binding isotherms fit reasonably well to the typical one-to-one binding model (Fig. 2). Removing either of the two D317 and D118 residues completely abolished the binding reaction, while the mutation of Y313 and R324 introduced much less serious reduction in binding affinity (Table 1). In particular, the Tyr-to-Ala mutation at position 313 resulted in a merely two-fold reduction in binding affinity. On the other hand, both the Y313A and the R324A mutation greatly reduced the favorable enthalpy of binding, which were largely compensated for by the favorable change in binding entropy.

**Figure 2:**
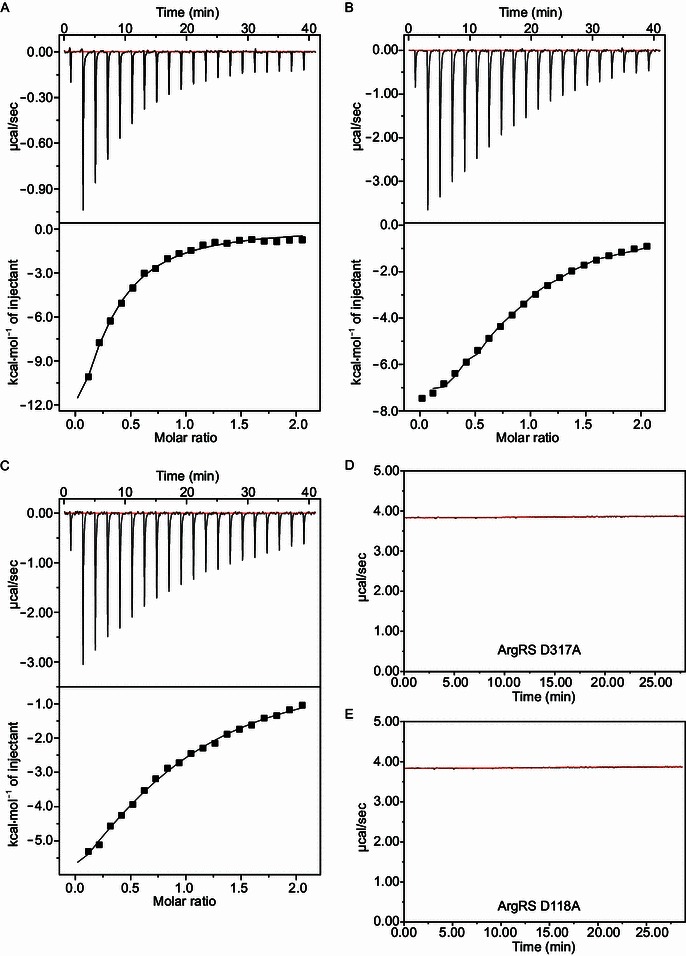
**Binding isotherms of WT and mutant ArgRS with L-arginine**. (A) Wild type; (B) Y313A; (C) R324A; (D) D317A; (E) D118A

**Table 1:** Binding thermodynamics of WT and mutant eArgRS with L-arginine

ArgRS	Stoichiometry (N)	*K*_a_ (M^-1^)	ΔG (kcal/mol)	ΔH (kcal/mol)	-T•ΔS (kcal/mol)
WT	0.69	2.15E4	−5.9	−16.0	10.1
Y313A	0.86	1.24E4	−5.4	−10.5	5.1
R324A	0.82	5.23E3	−4.8	−11.3	6.4
D317A	N/A				
D118A	N/A				

### The A20 recognition pocket and the Add1 domain

The N-terminal domain (NTD, Add1), a unique structural feature of ArgRS, plays a critical role of recognizing the identity elements of cognate tRNA. Phylogenetic analysis identified the nucleotide A20 as the major identity element (Liu et al., 1999), which is highly conserved in tRNA^Arg^ of most organisms with only a few exceptions including yeast. The N-terminal Add1 domain accommodates the A20 recognition pocket as found in the structures of ttArgRS and pyroArgRS (Shimada et al., 2001; Konno et al., 2009). The bottom of the pocket is formed by the antiparallel strands β3 and β4. Cavarelli et al. (1998) and Shimada et al. (2001) proposed a Tyr/Phe-Asn/Gln motif in the Add1 domain for A20 recognition. Both the primary structure and tertiary structure analyses confirmed that in eArgRS this motif was present as Y84–N82 (Fig. 3), indicating that A20 recognition is highly conserved in ArgRS.

**Figure 3:**
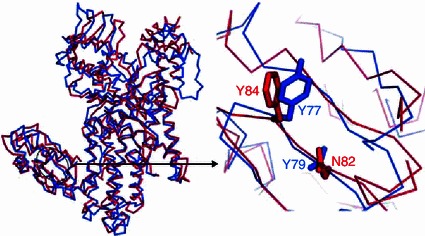
**Residues critical for recognizing the identity element A20: yeast (blue, PDB ID: 1BS2), eArgRS (red)**

Although the NTD did not undergo significant movement (relative to the rest of the enzyme) upon tRNA binding (Delagoutte et al., 2000), Shimada et al. noted that the overall orientation of NTD with respect to the rest of the enzyme in ttArgRS was markedly different from that of yArgRS (Shimada et al., 2001). The NTD of eArgRS adopted a different orientation from that of yArgRS as well. When superimposed based on the active site Cα atoms of ArgRS, the L-arginine ligands in yArgRS and in eArgRS aligned nearly perfectly (Fig. 4A). However, when the NTD Cα atoms served as the superposition standard, we observed large deviation between the active sites of both orthologues (Fig. 4B), which is independent of tRNA and L-arginine binding (Cavarelli et al., 1998; Delagoutte et al., 2000).

**Figure 4:**
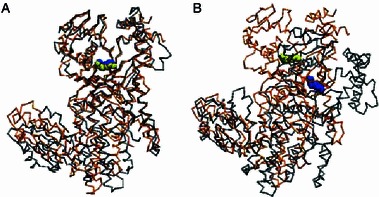
**Different NTD orientations with respect to the catalytic domain in eArgRS and yArgRS**. (A) yArgRS (gray, PDB ID: 1BS2) is superimposed to eArgRS (orange) according to the active site domain. The bound L-arginine molecules are depicted as blue (yArgRS) and yellow (eArgRS) vdW spheres. (B) yArgRS is superimposed to eArgRS according to the NTD

Difference in the NTD orientations of various ArgRSs most likely reflected the difference between the relative orientations of the tRNA identity element region and those of the acceptor stem region. We picked helix α1 and the strand β5 as representatives of the NTD and the active site respectively, and calculated the angle between these two motifs (ζ). The three reported yArgRS structures had an average ζ of 125.2˚ ± 0.8˚, while for eArgRS, ttArgRS and pyroArgRS, ζ were 120.0˚, 122.1˚ and 113.9˚, respectively. If we regard the archaic pyroArgRS as the most primitive enzyme in regard to evolution, it appears that ArgRSs of higher species tend to have larger ζ than those of lower ones in general. Nonetheless, the exact angle is highly species-dependent.

### The Ω-loop

The Ω-loop (A451–A457), connecting helices α15 and α16, acts as a molecular switch upon tRNA binding (Delagoutte et al., 2000). Using the residues spanning Ω-loop, α15 and α16 as superposition standard, the eArgRS Ω-loop structure aligned perfectly with the yArgRS-Arg complex (Table 2). The conformational change in this region upon tRNA binding manifests mainly as larger deviation of Cα atoms from eArgRS in α15 (Table 2).

**Table 2:** Cα RMSD between eArgRS-arginine structure and yeast structure

Secondary structure	yArgRS-tRNA-arginine	yArgRS-tRNA	yArgRS-arginine
H17(yeast)-α15	1.2 Å	1.3 Å	0.5 Å
H18(yeast)-α16	2.4 Å	2.4 Å	0.3 Å
Ω-loop	1.2 Å	1.2 Å	0.4 Å

Yao and co-workers measured the lifetimes of the tRNA-bound and free conformations of this α15-Ω-α16 region in eArgRS using ^19^F NMR spectroscopy (Yao et al., 2003, 2004). The interconversion between the bound and the free conformations proceeds in the intermediate to slow exchange regime of ^19^F NMR time scale. In the eArgRS structure, we observed a special structural feature that is absent in the yeast orthologue. R400 is in close contacts with both W446 and E396 (Fig. 5). While being stabilized by the salt bridge from E396, the guanidinium moiety of R400 adopts a conformation parallel to the indole ring of W446, where a cation-π interaction is likely in play. Adding to more constraints of W446, it is also surrounded by a series of non-polar side chains, which resembles the yArgRS structures. Sequence alignment shows that yArgRS also possesses these three residues on helices H15 and H17 (helices α13 and α15 in eArgRS): E424, K428 and W475 (Fig. 5). However, W475 of yArgRS locates too far from K428 to allow any direct contact. yArgRS without tRNA has E424 and K428 pointing away from the Ω-loop (Fig. 5B). The binding of tRNA to yArgRS results in a ~90˚ rotation of helix H15 and a downward shift by about one helix turn (Fig. 5C and 5D) (Delagoutte et al., 2000). If such conformational change is maintained in eArgRS upon tRNA binding, the W446-R400 cation-π interaction would be absent in the tRNA-bound form, which might in turn rationalize the slow exchange kinetic feature measured in ^19^F NMR experiment (Yao et al., 2004). That is, the W446-R400 interaction serves to lengthen the lifetime of the tRNA-free conformation. Enzymatic assays and preferably ^19^F-NMR binding kinetics study of yArgRS, and the eArgRS-tRNA complex structure are required to further confirm the role of this W446-R400-E396 triad in controlling the conformation of Ω-loop and the binding kinetics of cognate tRNA.

**Figure 5:**
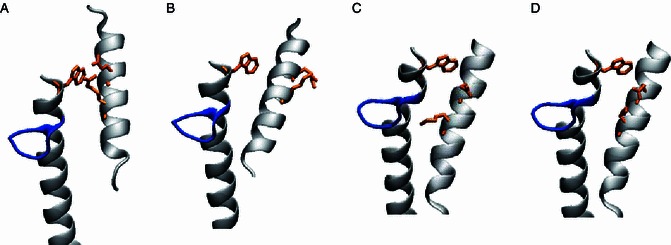
**Structural comparison of the triad W446-R400-E396** **in eArgRS and** **W475-K428-E424** **in yArgRS**. Comparison between the eArgRS-L-arginine structure (A) and the yArgRS-L-arginine structure (B–D, PDB ID: 1BS2, 1F7U, 1F7V) at the region of α15-Ω-α16 and α13 (H17-Ω-H18 and H15 in yeast). The Ω-loop, is highlighted in blue. The W446-R400-E396 triad of *E. coli* and the corresponding residues of yArgRS (W475-K428-E424) are highlighted in orange

## Discussion

In this study, we solved the crystal structure of *E. coli* arginyl-tRNA synthetase in complex with its substrate L-arginine, and carried out ITC studies of a series of mutant enzymes. In addition to providing more information regarding the mechanism of tRNA binding by ArgRS, our study shed considerable light on the less understood arginine recognition.

Although several residues in the arginine binding pocket are highly conserved among ArgRS, or even among many aaRS’s, our mutational study showed that some residues may in fact be non-essential for achieving high binding affinity. The elimination of electrostatic interactions by mutating either D317 or D118 abolished the binding of L-arginine, while the enzyme without Y313 or R324 retained some of the wild type’s ligand binding ability. In contrast to the small changes in binding free energy, a closer look at the thermodynamic data of the Y313A and R324A mutants revealed much greater changes in binding enthalpy and binding entropy from those of the wild type. The well-documented enthalpy-entropy compensation manifested in both mutants (Dunitz, 1995; Lemieux, 1996; Perozzo et al., 2004). R324 does not make direct contact with the bound arginine, but serves to lock the side chain of D118 at a favorable rotameric conformation (Fig. 1C). Without the salt bridge from R324, D118 was allowed to sample a much larger configurational space and might be adopting a less favorable conformation upon the binding of arginine, giving rise to a 4.7 kcal/mol drop in binding enthalpy. Such large decrease of favorable (negative) binding enthalpy was well compensated by a 3.7 kcal/mol favorable (positive) change in T∆S. That is, L-arginine is so tightly bound in the active site that any destabilization of the highly specific interactions increases the overall degrees of freedom.

A comparison with the yArgRS structure without arginine showed that the highly conserved Y313 was solvent-exposed and hydrogen bonded to the main chain carbonyl oxygen atom of W162 in the absence of arginine. The binding of arginine induced the rotation of the Cα-Cβ bond in Y313 and the Y313-W162 H-bond switched to the Y313-L-arginine H-bond. Mutation of W162 to alanine diminished the catalytic activity of eArgRS (Zhang et al., 1998). Using ^19^F NMR spectroscopy, Yao et al. showed that W162 was involved in arginine binding (Yao et al., 2003). Since only the main chain atoms participate in the Y313-W162 hydrogen bond, the side chain of W162 may be involved in maintaining a favorable orientation of the backbone carbonyl group towards the Y313 side chain hydroxyl group. Presumably, the Y313A mutation should introduce negligible change in binding enthalpy, given that Y313 is always involved in hydrogen bonding prior and after the binding of L-arginine. However, the drop of ∆H in the Y313A mutant from that of the wild type was as large as 5.5 kcal/mol. A closer look at the active site of the eArgRS-L-arginine complex revealed a closed loop of hydrogen bonding moieties, consisting of L-arginine, Q341 and Y313 (Fig. 6A). Y313 accepts a hydrogen bond from the guanidinium group of L-arginine and donates a hydrogen bond to the amide carbonyl group of Q341. Q341 in turn donates a hydrogen bond to the carbonyl group of L-arginine. The cooperative effect renders this hydrogen bond network highly favorable energetically. In the Y313A mutant however, Q341 would participate in only one hydrogen bond with the carbonyl group of L-arginine. It is not uncommon to have an enthalpy difference as large as 5 kcal/mol between the presence and absence of cooperative hydrogen bonding (Elrod and Saykally, 1994). On the other hand, the hydrogen bond loop of L-arginine-Q341-Y313 imparted restriction to the mobility of L-arginine. The removal of Y313 from the loop significantly increased the degrees of freedom for L-arginine. As a result, the favorable change in T∆S nearly canceled the unfavorable change in ∆H, leaving the difference in ∆G at only 0.5 kcal/mol.

**Figure 6:**
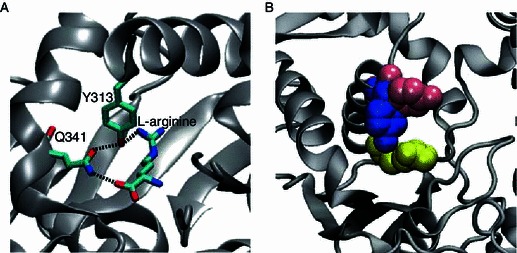
**Thermodynamic and kinetic roles of residues in the L-arginine binding site**. (A) Y313, Q341 and L-arginine comprise a cooperative ring of hydrogen bonds (black dashed line). (B) Y313 locks the bound arginine (yellow) in the active site. Upon the binding of arginine, the phenol ring of Y313 swings from a horizontal orientation (pink) to a vertical downward orientation (blue), which shuts the active site. The coordinates of the phenol ring in the absence of arginine (pink) were taken from the corresponding residue of Y347 in yeast structure (PDB ID: 1F7V)

Although Y313 is strictly conserved among GlnRS, GluRS, TyrRS, TrpRS and ArgRS, this tyrosine residue assumes different roles in different aaRS’s. In particular, Y313 of eArgRS acts as a gate-keeper: keeping the binding pocket open in the absence of arginine via the Y313-W162 H-bond, while locking the bound arginine in position after rotameric rotation. Y313 contributes more in the kinetics regime of arginine binding rather than in the regime of binding thermodynamics (affinity)—keeping the substrate in the active site sufficiently long for catalysis to take place (Fig. 6B). Further NMR binding kinetics study and enzymatic assays of the mutants are required to confirm our conclusion based on crystal structures.

## Materials and Methods

### Gene expression and protein purification

We used the plasmid containing ArgRS gene derived from *E. coli* as a template to amplify the ArgRS gene, using the Primer5 software (http://www.uea.ac.uk/~e130/Primer5.htm) to design the up primer 5′-CGGGATCCATGAATATTCAGGCTCTTCTCTC-3′ and the down primer 5′-CCCAAGCTTTTACATACGCTCTACTGTCTC-3′. The gene was inserted into another plasmid vector pET-28a-SUMO. We transformed the pET28a-SUMO/ArgRS plasmid into the expression bacterial *E. coli* BL21 (DE3) (Novagen, Madison, WI). Protein expression was induced with 0.2 mmol/L isopropyl β-D-1-thiogalactopyranoside (IPTG) at an optical cell density (OD_600_) of ~0.8, followed by incubation at 16°C for 20 h. Cell pellet was sonicated with lysis buffer (20 mmol/L Tris-HCl pH 7.5, 300 mmol/L NaCl, 10 mmol/L β-mercaptoethanol, 1 mmol/L PMSF). The lysate was centrifuged at 18,000 rpm for 30 min. The supernatant was incubated with Ni^2+^-NTA affinity resin (Qiagen) for 3 h at 4°C, then the resin was eluted with an imidazole gradient up to 200 mmol/L imidazole and the target protein was eluted with 100 mmol/L imidazole. The protein was dialyzed overnight against a reservoir solution of 20 mmol/L Tris-HCl (pH 7.5) and 300 mmol/L NaCl in the presence of Ulp1 protease (made in house) which cleaved the N-His-SUMO tag from the target protein (enzyme:protein = 1:30 *w*/*w*). The released His-SUMO tag was removed by a second round of Ni^2+^-NTA chromatography. The protein was further purified by size exclusion chromatography using HiLoad 16/60 Superdex 200 column (GE Healthcare) in gel-filtration buffer (20 mmol/L Tris-HCl pH 7.5, 300 mmol/L NaCl). The volume of the collected protein sample was reduced to reach a final concentration of 10 mg/mL.

### Crystallization and data collection

Initial crystallization was screened by several commercial kit, such as crystal I/II and Index (Hampton Research) and crystals were obtained in the reservoir containing 100 mmol/L Tris-HCl pH 8.5, 200 mmol/L ammonium acetate, 25% PEG-3350. We mixed the protein sample with its substrate including 2 mmol/L L-arginine, 5 mmol/L ATP. At last, the best crystal was grown at 16°C by sitting drop diffusion method against a reservoir solution of 50 mmol/L HEPES (pH 7.2), 100 mmol/L sodium acetate, 22% PEG-3350. We mixed the 1 μL reservoir with 1 μL protein solution which contained 10 mg/mL ArgRS, 2 mmol/L L-arginine, 5 mmol/L ATP, 10 mmol/L MgCl_2_. Crystal grew in two days and its shape was rectangle with sharp edge. Ethylene glycol was used as cryoprotectant. We first soaked the crystal in the 10% ethylene glycol and then transferred the crystal into a higher ethylene glycol concentration up to 20%. Finally, the crystal was preserved in the liquid nitrogen.

For data collection, Diffraction data was collected at the beamline BL17U of Shanghai Synchrotron Radiation Facility (SSRF) using an ADSQ315 CCD detector. Data were processed with HKL2000 (Otwinowski and Minor, 1997). The crystal structure of ArgRS belonged to the space group C_2_. The partial coordinates of yeast ArgRS (PDB 1BS2) (Cavarelli et al., 1998) as ensemble 1 and the structure 3GDZ as ensemble 2 were used as search models for ArgRS using the program Phaser from CCP4 package (Read, 2001). The initial model was built using the rigid body refinement with Phenix.refine in the Phenix program (Adams et al., 2010). Subsequent refinement was carried out with alternating cycles of manual refitting and building under 2Fo-Fc and 1Fo-Fc electron density map in Coot (Emsley and Cowtan, 2004) and using XYZ coordinates and individual B factors in the Phenix.refine program (Adams et al., 2010). The *R*-working and *R*-free dropped to 23.9% and 26.5% for all data from 30 Å to 2.6 Å. The final structure was checked for geometrical correctness with PROCHECK (Laskowski et al., 1993). Data collection and refinement statistics are summarized in Table 3. The atomic coordinates and the structure factor have been deposited in the Protein Data Bank (PDB code: in submission). All structural figures were generated using PyMOL (http://www.pymol.org) and VMD (Humphrey et al., 1996).

**Table 3:** Statistics of data collection and model refinement

**Data collection**	
Space group	*C2*
Unit cell parameters (Å)	a = 118.60, b = 94.14, c = 62.72; α = γ = 90°, β = 110.5°
Resolution range (Å)	50–2.60 (2.69–2.60)
No. of total reflections	133676 (12309)
No. of unique reflections	20334 (2018)
*I*/*σ*	13.6 (4.8)
Completeness (%)	99.5 (99.4)
*R*_merge_ (%)^a^	12.0 (65.1)

**Structure refinement**	
Resolution (Å)	30–2.6
*R*_cryst_/*R*_free_ (%)^b^	23.9/26.5
r.m.s. deviations	
Bond bonds (Å)	0.003
Bond angles (º)	0.750

**Ramachandran plot**	
Favored region (%)	93.5
Allowed region (%)	4.5
Outlier region (%)	2.0

**No. of atoms**	
Protein	4248
Water	30
Ligand	12

^a^*R*_merge_ = ∑|I_i_ − I_m_|/∑I_i_, where I_i_ is the intensity of the measured reflection and I_m_ is the mean intensity of all symmetry related reflections

^b^*R*_cryst_ = Σ||F_obs_| − |F_calc_||/Σ|F_obs_|, where F_obs_ and F_calc_ are observed and calculated structure factors

*R*_free_ = Σ_T_||F_obs_| − |F_calc_||/Σ_T_|F_obs_|, where T is a test data set of about 5% of the total reflections randomly chosen and set aside prior to refinement

Numbers in parentheses represent the value for the highest resolution shell

### Isothermal titration calorimetry study of the wild type and mutant ArgRS

We designed four ArgRS mutants including D118A, D317A, Y313A, R324A based on ArgRS structure. The mutation was introduced into pET22b/ArgRS using the site-directed mutagenesis and purified these proteins with the same method as described previously. The formation constant and thermodynamic parameters for the inclusion of Arginine in ArgRS were measured by the titration calorimetry method by using an ITC MicroCal 200 (GE Life Sciences). All solutions were prepared in a 200 mmol/L Tris-HCl pH 7.5, 300 mmol/L sodium chloride. A solution (0.25 mmol/L) of ArgRS was placed in the sample cell, and a 2.5 mmol/L solution of Arginine was added in a series of 20 injections, the heat evolved was recorded at 25°C. The heat of injecting the Arginine into a neat buffer solution is nearly zero. The data were analyzed and the binding isotherm was fitted to a single-site model in the ORIGIN 7.0 software (GE Life Sciences).

## Electronic supplementary material

Below is the link to the electronic supplementary material.

Supplementary material 1 (PDF 463 kb)

## Abbreviations

AMP-CPP: adenosine 5′-α,β-methylene triphosphate
ArgRS: arginyl-tRNA synthetase
ATP: adenosine triphosphate
HEPES: 4-(2-hydroxyethyl)-1-piperazine ethanesulfonic acid
ITC: isothermal titration calorimetry
Ni^2+^-NTA: Ni^2+^ nitrilotriacetic acid
NTD: N-terminal domain
PMSF: phenylmethanesulfonyl fluoride
