# Supplementary material for: Crystal structure of E. coli arginyl-tRNA synthetase and ligand binding studies revealed key residues in arginine recognition
Source: Protein Cell. 2014 Jan 30;5(2):151–9. doi: 10.1007/s13238-013-0012-1 (PMC3956977; doi:10.1007/s13238-013-0012-1)
Supplement: Supplementary file 1 [file 13238_2013_12_MOESM1_ESM.pdf]

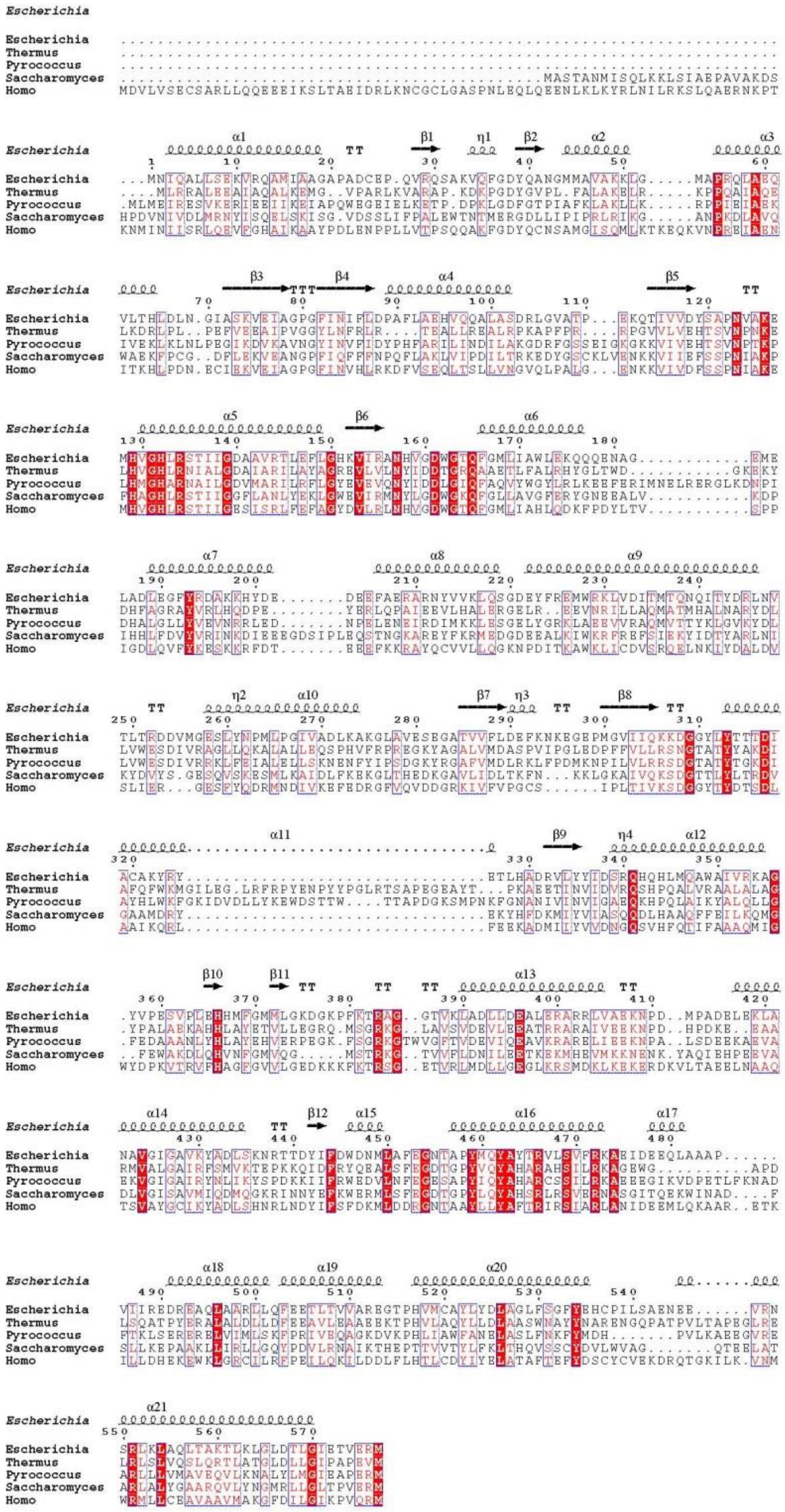

Sequence alignment for ArgRSs from various organisms. Sequence alignment for ArgRSs from various organisms: Escherichia, E.coli; Thermus, Thermus thermophilus; Pyrococcus, Pyrococcus horikoshii; Saccharomyces, Saccharomyces cerevisiae; Homo, Homo sapiens. The secondary structure assignment is also displayed based on the eArgRS structure. Elements of secondary structure are indicated by: α for α-helix, β for β-sheet, η for 310 helix, TT for turn. Residue numbering is shown above the sequence of eArgRS. The alignment was carried out using the program CLUSTAL W. This figure was produced with the program ESPript.
